# Graded Epidural Anaesthesia for Caesarean Section in the Presence of an Untreated Spetzler-Martin Grade V Cerebral Arteriovenous Malformation With Radiological Progression Over Six Years: A Case Report

**DOI:** 10.7759/cureus.115598

**Published:** 2026-09-01

**Authors:** Dasara Nongbet, Newstar Syiemiong, Neha Rawat, Thongam S Devi, Gareth Y Lyngwa

**Affiliations:** 1 Anaesthesiology, North Eastern Indira Gandhi Regional Institute of Health and Medical Sciences, Shillong, IND; 2 Radiodiagnosis, North Eastern Indira Gandhi Regional Institute of Health and Medical Sciences, Shillong, IND

**Keywords:** cerebral arteriovenous malformation, graded epidural anaesthesia, informed refusal, pregnancy complications, spetzler-martin grade v

## Abstract

Cerebral arteriovenous malformations (AVMs) are rare during pregnancy, when the haemodynamic and hormonal changes of gestation may increase the risk of rupture. Lesions that are large, located in eloquent regions, and drained by deep venous channels represent a surgically high-risk subtype, carry substantial surgical morbidity, and are frequently managed conservatively when unruptured. We describe a 33-year-old G3P1L1A1 parturient at 36 weeks and six days of gestation with an untreated right parieto-temporo-occipital AVM, who declined intervention across three encounters and underwent an elective lower-segment Caesarean section (LSCS) with bilateral tubal ligation. The anaesthetic approach chosen was a graded epidural technique: fractionated bupivacaine boluses with real-time haemodynamic monitoring between increments. This allowed the rate of sympathectomy to be controlled, avoiding the abrupt sympathectomy associated with a subarachnoid block. Carbetocin was used as the uterotonic agent. Levetiracetam was continued perioperatively. A healthy male infant was delivered, and the mother was discharged neurologically intact on day seven, with no AVM rupture, seizure, or new deficit.

## Introduction

Cerebral arteriovenous malformations (AVMs) are congenital vascular anomalies in which dysplastic vessels shunt arterial blood directly into the venous circulation, bypassing the capillary bed. Their prevalence is estimated at 10 to 18 per 100,000 in the general population [1, 2], with a mean age at diagnosis of 31.2 years, which falls within the reproductive years [3]. The annual risk of haemorrhage from an unruptured AVM is estimated at 2% to 4% [4]. Whether pregnancy independently increases this risk remains contested; estimates in pregnant patients cluster towards the upper end of the reported range, at approximately 3.5% [5]. The consequences of rupture during pregnancy are severe, with substantial maternal and foetal mortality reported in both historical and contemporary series [6, 7].

The Spetzler-Martin (S-M) grading system classifies AVMs according to three parameters: nidus size (less than 3 cm = one point; 3 to 6 cm = two points; greater than 6 cm = three points), eloquence of the adjacent brain (eloquent = one point; non-eloquent = zero points), and venous drainage pattern (deep = one point; superficial = zero points), yielding a maximum score of five [8]. Grade V lesions, the highest possible grade, occupy the eloquent cortex, are large, and drain via deep venous channels; they carry substantial surgical morbidity, are rarely cured by embolisation alone, and are frequently managed conservatively when unruptured [9].

Neuraxial anaesthesia is generally preferred over general anaesthesia for lower-segment Caesarean section (LSCS) in appropriately selected parturients with cerebral AVMs because it avoids the laryngoscopy-related blood pressure surge, the cerebral vasodilatory effects of volatile agents, positive-pressure ventilation, and emergence hypertension, thereby limiting factors that may increase pressure across an unprotected nidus or raise intracranial pressure (ICP) [10]. Published neuraxial experience in this population is, however, largely confined to lesions that have undergone prior embolisation or radiosurgery [11]. To our knowledge, there is no published report of a graded epidural technique in an untreated high-grade lesion with established mass effect; the one comparable untreated Grade V case was managed under general anaesthesia [12].

We report the management of a 33-year-old parturient with a confirmed S-M Grade V cerebral AVM who declined interventional treatment during three documented encounters and underwent an LSCS under graded epidural anaesthesia at a tertiary referral teaching hospital, achieving a favourable outcome.

## Case presentation

A 33-year-old G3P1L1A1 parturient at 36 weeks and six days of gestation by dates (38 weeks and five days by scan) was admitted for an elective Caesarean section planned at 37 weeks. She weighed 80 kg and was 153 cm tall, with a BMI of approximately 34 kg/m². She had been diagnosed with a cerebral AVM in the right parieto-temporo-occipital region at 19 years of age and had not received definitive treatment in the 14 years since diagnosis. There was no personal or family history of diabetes mellitus, hypertension, bronchial asthma, chronic obstructive pulmonary disease, tuberculosis, or prior surgery. She was a non-smoker with no alcohol or tobacco use. Her obstetric and neurological histories together formed the basis of the conservative management strategy. Her first pregnancy at 19 years resulted in a full-term normal vaginal delivery of a healthy girl, now 14 years old. Neurological symptoms first appeared two weeks postpartum and were managed symptomatically, followed by an asymptomatic interval of four years. When the headaches recurred, embolisation was advised at a higher centre, but the patient declined. Her second pregnancy, eight years later, brought a recurrence of symptoms and ended in a spontaneous abortion at three months, managed with dilatation and curettage. A digital subtraction angiogram (DSA) was performed afterwards, but the COVID-19 lockdown precluded further intervention. Embolisation was again proposed during a review three years later but was again declined. She conceived the current, third pregnancy five years after the abortion and attended regular antenatal care.

At 29 weeks and five days of gestation (31 weeks and four days by scan), she was admitted for one week with decreased foetal movements, a frontoparietal headache (right > left), and insomnia. A multidisciplinary approach was adopted throughout the remainder of the antenatal course. Neurology commenced levetiracetam at a dosage of 500 mg three times daily. Neurosurgery recommended coil embolisation, which the patient again declined; this decision was documented as informed refusal, providing the medicolegal basis for conservative management. She was discharged for continued outpatient antenatal follow-up.

Preoperative assessment

At her antenatal review at 36 weeks and six days, immediately before the planned admission for delivery, she had no new neurological complaints, seizures, headache, visual disturbance, or signs of raised ICP. There was no bleeding or leakage per vagina and no abdominal pain. Foetal movements were normal. The patient was conscious and orientated, with a Glasgow Coma Scale score of 15/15 (E4V5M6); pallor, icterus, cyanosis, clubbing, lymphadenopathy, and oedema were absent. Her blood pressure was 110/62 mmHg, heart rate was 80 beats per minute, and peripheral oxygen saturation (SpO₂) was 96% on room air. The respiratory system was clear bilaterally; cardiovascular examination revealed normal heart sounds with no murmurs. Central nervous system examination demonstrated no focal neurological deficit, with power of 5/5 in all four limbs, and these findings were formally documented as the neurological baseline for postoperative comparison. Abdominal examination revealed a term-sized, relaxed, cephalic uterus with a foetal heart rate of 142 beats per minute. All preoperative investigations were completed before surgery and showed no clinically significant abnormalities (Table 1).

**Table 1:** Preoperative laboratory results. INR: International normalised ratio; PT: Prothrombin time; APTT: Activated partial thromboplastin time; TSH: Thyroid-stimulating hormone.

Investigation	Value	Reference range
Haemoglobin	12.6 g/dL	12-18 g/dL
WBC	12.0 × 10³/µL	4-11 × 10³/µL
Platelets	340 × 10³/µL	150-400 × 10³/µL
INR	0.99	<1.5
PT	11.6 s	11-13.5 s
APTT	22.5 s	21-36 s
Sodium	133 mmol/L	135-145 mmol/L
Potassium	3.88 mmol/L	3.5-5.5 mmol/L
Urea	15 mg/dL	17-45 mg/dL
Creatinine	0.7 mg/dL	0.6-1.4 mg/dL
TSH	0.81 mIU/L	0.4-4.0 mIU/L

Serial foetal surveillance throughout the pregnancy confirmed a single viable foetus with active movements. The anomaly scan at 20 weeks and four days demonstrated a normally formed foetus and a normal foetal echocardiogram. Growth scans at 25 weeks and two days and at 36 weeks and six days documented appropriate foetal growth, with no evidence of uteroplacental insufficiency.

Three serial radiological investigations spanning six years constitute the primary imaging contribution of this report. A six-vessel cerebral DSA was performed via the transfemoral route when the patient was 27 years old. The reported impression was a large, diffuse, high-flow AVM in the right posterior temporo-occipital and peritrigonal regions, with a nidus measuring 3.5 cm in diameter, classified as S-M Grade III.

A 3-tesla brain MRI scan was performed when the patient was 30 years old and revealed a right temporo-occipital AVM with a nidus measuring 8.6 × 5.8 × 5.1 cm; the maximum reported dimension was more than twice the measurement reported on the previous DSA. No formal S-M grade was assigned in that report.

A further 3-tesla brain MRI scan was performed during the current pregnancy, at 30 weeks of gestation and 33 years of age. The scan demonstrated a nidus measuring 9.4 × 6.6 × 4.2 cm in the right parieto-temporo-occipital lobe, classified as S-M Grade V. Importantly, a contralateral midline shift of approximately six millimetres was identified. This was a new finding, absent from the previous MRI, indicating the development of significant mass effect during the intervening three years, as shown in Figures 1A-1B.

**Figure 1:**
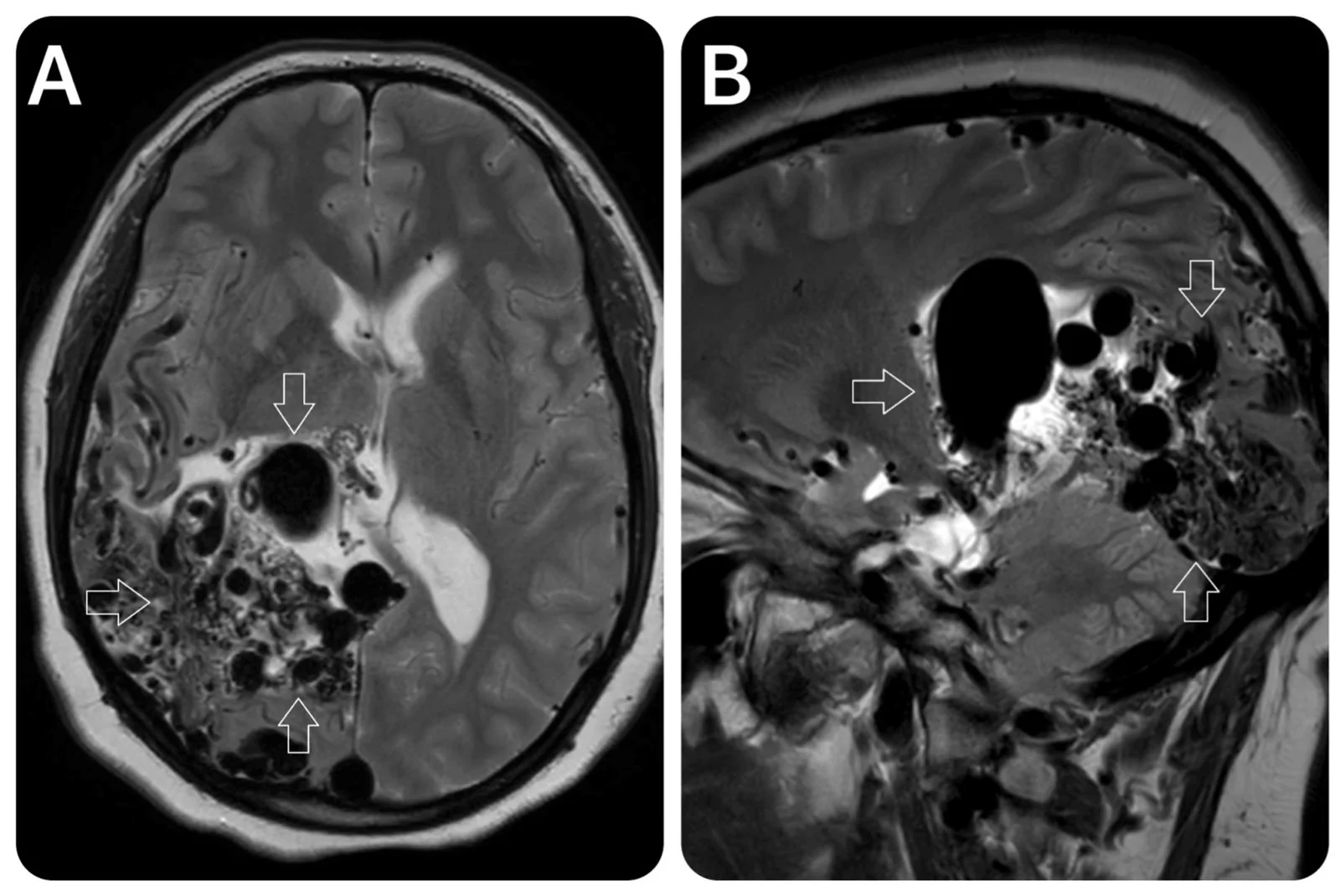
Brain MRI showing a Spetzler-Martin Grade V arteriovenous malformation with a 6 mm midline shift. (A) Axial and (B) sagittal T2-weighted images of the brain show a vascular nidus (arrows) in the right parieto-occipital lobe, consisting of multiple dilated and tortuous vessels that appear as flow voids.

Anaesthetic management

The predefined anaesthetic plan was established preoperatively and reiterated in the operating theatre. Preoperative preparation included IV access with two large-bore (18G) cannulae, one in each hand, and standard ASA monitoring (continuous ECG, non-invasive blood pressure, SpO₂, and capnography); an invasive arterial line was also secured in the left radial artery under aseptic precautions. Left lateral uterine displacement was performed; levetiracetam 500 mg IV was immediately available; IV benzodiazepine was kept at the bedside for breakthrough seizures; and an emergency general anaesthetic plan was prepared. Two units of packed red cells were crossmatched and held in the blood bank.

Graded epidural anaesthesia was selected over a single-shot spinal. The epidural catheter was placed with the patient in the left lateral decubitus position. The L3-L4 interspace was identified using Tuffier’s line and palpation. The epidural space was identified at a depth of 5 cm using the midline approach and loss of resistance to saline, and the catheter was secured at 10 cm at the skin. A 3-mL test dose of plain 2% lignocaine was administered to rule out subarachnoid placement, and adrenaline was omitted because of the underlying pathology.

A graded protocol was used, consisting of five 3-mL aliquots of 0.5% bupivacaine administered at five-minute intervals (total 15 mL, 75 mg), with continuous haemodynamic monitoring between doses. The sensory level was assessed by pinprick at 0, 5, 10, 15, and 20 minutes. A bilateral, symmetrical sensory block to the T4 dermatome was confirmed before skin incision, which was performed 30 minutes after the first epidural dose. Mean arterial pressure (MAP) ranged from 70 to 89 mmHg intraoperatively against a baseline of 81 mmHg, remaining within 10% of baseline at all time points except one. A transient nadir of 70 mmHg at 35 minutes, representing a 13.6% fall below baseline, prompted the commencement of a phenylephrine infusion (50 µg/mL) at 0.3 µg/kg/min (24 µg/min at 80 kg). The infusion was progressively weaned to 0.2 and then 0.1 µg/kg/min as haemodynamics stabilised, giving a total intraoperative dose of approximately 440 µg over 25 minutes. Blood pressure did not fall below 80% of baseline at any point. Haemodynamic trends are presented in Figures 2-3.

**Figure 2:**
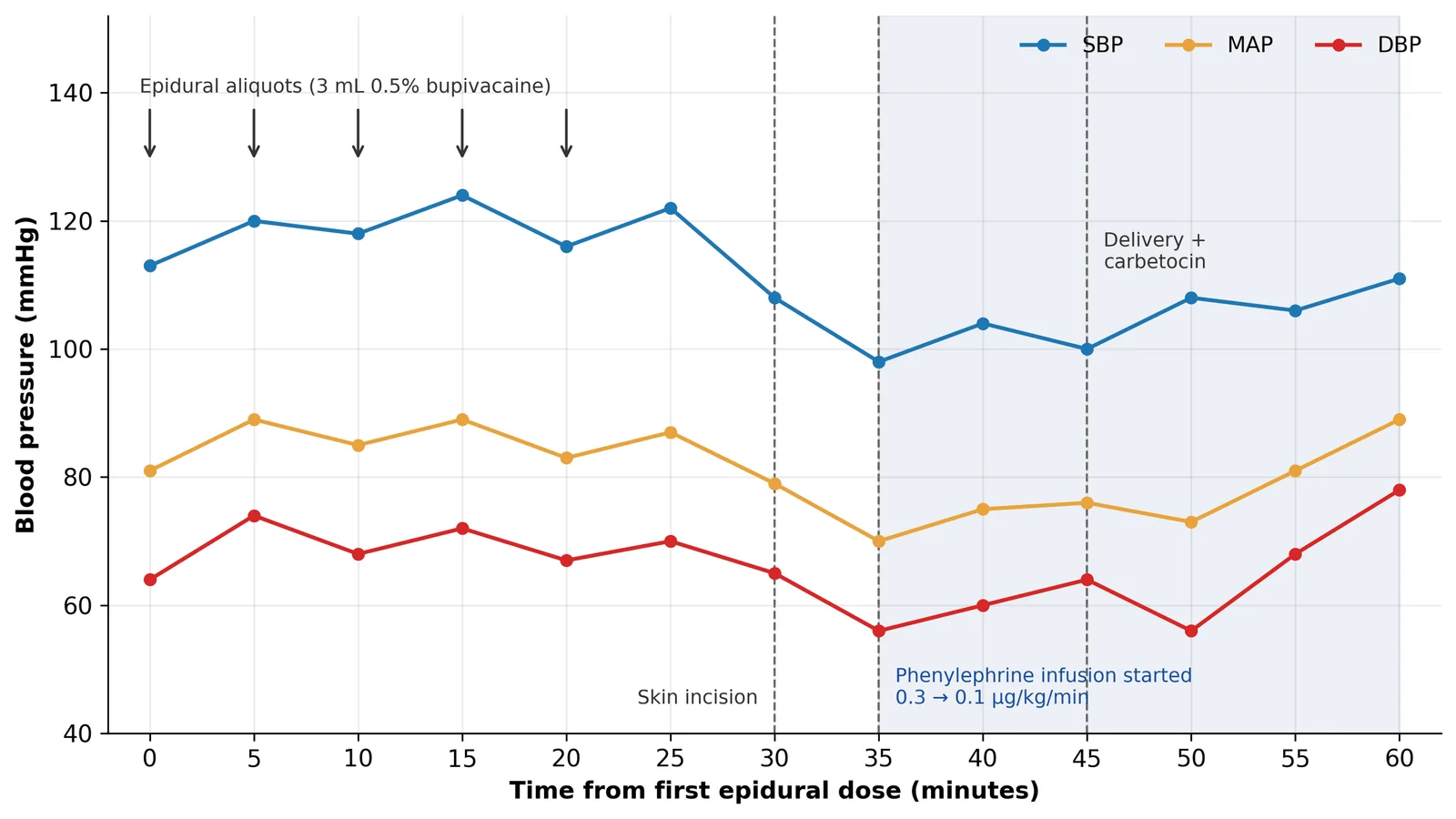
Blood pressure trends. SBP: Systolic blood pressure; DBP: Diastolic blood pressure; MAP: Mean arterial pressure.

**Figure 3:**
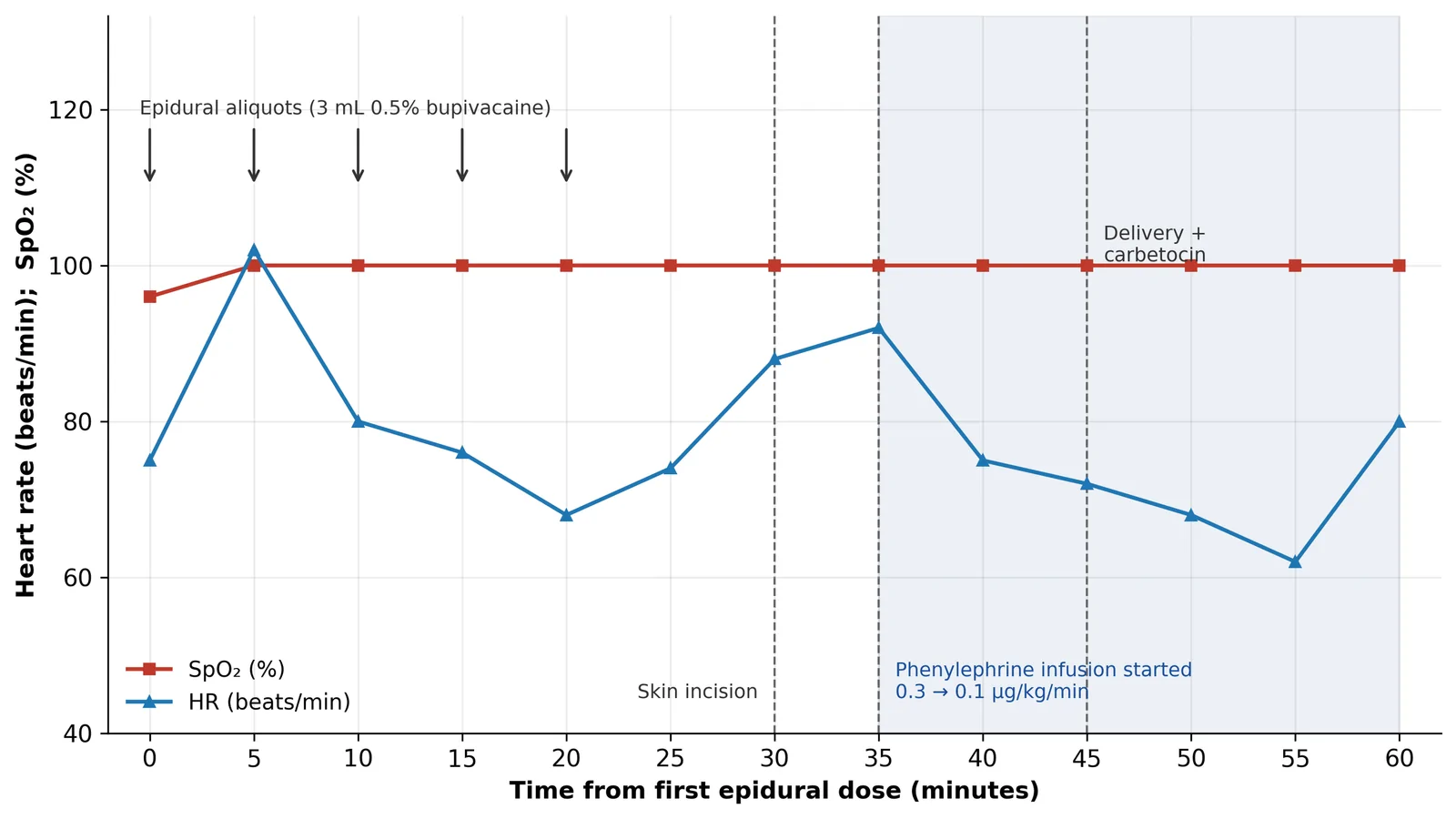
Heart rate and oxygen saturation. HR: Heart rate; SpO₂: Peripheral oxygen saturation.

Baseline intraoperative monitoring documented a blood pressure of 113/64 mmHg (MAP 75 mmHg), a heart rate of 81 beats per minute, and an SpO₂ of 96%. Supplemental oxygen at 3 litres per minute via nasal prongs was maintained continuously; SpO₂ normalised to 100% within minutes of the positional adjustment and remained at 99% throughout the intraoperative period.

A healthy male infant was delivered in the vertex presentation at 45 minutes, weighing 3.24 kg, with Apgar scores of 8 at one minute and 9 at five minutes. Carbetocin 100 µg was then administered slowly intravenously as the uterotonic of choice, and bilateral tubal ligation was performed. No neonatal resuscitation was required, and no neonatal intensive care admission was needed. Estimated blood loss was approximately 450 mL, and urine output was 200 mL.

The patient was transferred to the ICU immediately postoperatively for 48 hours. Monitoring included continuous ECG, non-invasive blood pressure, and SpO₂, together with neurological observation (GCS, pupils, speech, and limb power) against the documented preoperative baseline. A postoperative epidural infusion of 0.125% bupivacaine at 5 mL/hour provided continuous analgesia, with a Numeric Rating Scale (NRS) score ≤ 4 throughout, limiting pain-related catecholamine surges. Levetiracetam 500 mg IV three times daily was continued without interruption, and a haemodynamic target of MAP > 65 mmHg was maintained. She remained stable throughout, with no seizures, AVM-related complications, or new neurological deficits.

She then stepped down to the gynaecology high-dependency unit and was discharged on day seven with a blood pressure of 107/70 mmHg, heart rate of 92 beats per minute, SpO₂ of 97% on room air, GCS score of 15/15, an intact wound, and no new neurological deficits. Discharge medications included levetiracetam 500 mg three times daily, iron and folate, calcium, and analgesia. Follow-up was arranged with Gynaecology, Neurology, Interventional Radiology for AVM treatment planning, and Neurosurgery.

## Discussion

This case contributes to the obstetric anaesthesia literature across three distinct dimensions of novelty. First, the lesion was an untreated S-M Grade V cerebral AVM with documented mass effect and a 6 mm midline shift. Second, a protocolised graded epidural technique was used. Third, six-year serial radiological documentation provides the most detailed longitudinal AVM growth series for an unruptured lesion in the obstetric anaesthesia literature.

Isikkent D et al. reported two parturients with cerebral AVMs managed with neuraxial techniques: a combined spinal-epidural (CSE) with a single intrathecal bolus of 0.75% bupivacaine (12 mg) combined with morphine and fentanyl in a 27‑year‑old with a 2.9 cm occipital AVM previously embolised; and a standard fractionated epidural with invasive arterial line monitoring in a parturient with a basal‑ganglia‑region AVM earlier treated by gamma knife radiosurgery [11]. Both patients had undergone prior definitive treatment that reduced but did not obliterate the AVM, and both deliveries were planned. The present case extends that literature, as the lesion was untreated for 14 years from diagnosis and was S-M Grade V with a 6 mm midline shift. The anaesthetic technique was a graded epidural protocol using 3 mL aliquots of bupivacaine with 5-minute waits between doses. Unlike single-bolus CSE, this approach allowed the anaesthetist to titrate the speed of sympathetic blockade rather than simply its depth. The six‑year longitudinal radiological series provides imaging‑based evidence that cerebral AVMs can change, a phenomenon previously undocumented in the obstetric anaesthesia literature.

The Coskun report sits closest to ours in the literature. A 24-year-old primigravida presented at 16 weeks with a sudden headache and was found to have a giant S-M Grade V cerebrothalamic AVM; treatment was deferred when she declined pregnancy termination as a precondition for endovascular therapy, and she returned in spontaneous labour at 40 weeks for emergency Caesarean delivery under general anaesthesia with invasive arterial monitoring [12]. The lesion grade and absence of definitive treatment parallel our case, but the basis for non-treatment differed: our patient repeatedly declined embolisation itself across three encounters over 14 years, independent of pregnancy, whereas the Coskun patient’s treatment was deferred once, contingent on a termination decision she was unwilling to make. The anaesthetic approach also differed. Coskun D et al. chose general anaesthesia for tighter haemodynamic control; we chose graded epidural anaesthesia, which gave us direct control over the pace of the sympathetic block and avoided the laryngoscopy surge, volatile-agent vasodilation, and hypertensive rebound during emergence. Ours is, in the published literature, the first neuraxial case to sit alongside the Coskun report [12].

The case for graded epidural over single-shot spinal rests on one key factor: control. The AVM nidus has no capillary bed; therefore, changes in arterial pressure are transmitted directly to the venous side without buffering [10]. The speed of change matters as much as its magnitude. A single-shot spinal removes control from the moment the dose is administered. The sympathectomy that follows is rapid and unmodifiable. Hypotension triggers a baroreceptor reflex; overcorrection causes the pressure to rebound. This oscillation is inherent to the technique. A graded epidural keeps control in the anaesthetist’s hands. Small aliquots of bupivacaine are administered, followed by haemodynamic assessment after each one, and vasopressors are titrated before the next dose, keeping MAP within 10% of baseline.

The postoperative period carries its own haemodynamic risks, and the epidural catheter addressed them directly. Bupivacaine 0.125% at 5 mL/hr through the same catheter provided continuous analgesia throughout the 48-hour ICU stay, an advantage specific to the epidural technique and not replicable with either single-shot spinal or general anaesthesia. Pain triggers sympathoadrenal activation, raising heart rate, blood pressure, and cardiac output in a pattern that directly increases nidus shear stress. The epidural infusion blunts this response without the disadvantages of systemic opioids, which cause respiratory depression, hypercapnia, cerebral vasodilation, and increased ICP, precisely the physiological changes this patient’s Grade V AVM could least afford [10].

The choice of uterotonic was based on the same haemodynamic principles that governed the anaesthetic technique. Carbetocin 100 µg, administered as a slow intravenous injection, was used. Carbetocin is a synthetic, long-acting oxytocin analogue that causes effective and sustained uterine contractions after a single dose, without the vasoconstrictive surge of ergometrine or the reflex tachycardia and vasodilation that can occur with rapid oxytocin boluses. Its stable haemodynamic profile makes it the most appropriate uterotonic in this clinical context.

The six-year radiological series documents something rarely captured in the literature: an untreated AVM growing from approximately 3.5 cm in 2019 to over 9.4 cm by 2025, progressing from Grade III to Grade V. This information is relevant to the anaesthetist. A lesion that is enlarging, acquiring mass effect, and developing a midline shift is progressively less tolerant of haemodynamic fluctuations, which is why the controlled, incremental approach described here became increasingly important as the pregnancy advanced. The trajectory also carries a clear message for counselling women of reproductive age: definitive treatment before pregnancy is preferable to managing an expanding Grade V lesion during gestation [9, 13]. Growth is not universal among AVMs during pregnancy, and serial imaging surveillance remains informative on a case-by-case basis.

Maintaining uninterrupted antiepileptic prophylaxis with levetiracetam throughout the perioperative period until discharge was essential for maternal and foetal safety. Perioperative seizures are especially dangerous in this context because each ictus produces acute physiological stress, increased ICP, hypoxaemia, hypercapnia, and cardiovascular instability, all of which can precipitate haemorrhage in an unprotected Grade V AVM. Uninterrupted antiepileptic cover was therefore essential. The contrast with Isikkent’s index case is instructive: that patient discontinued her anticonvulsants after starting antenatal care and subsequently developed a constellation of neurological and visual symptoms culminating in seizures [11], underscoring why uninterrupted antiepileptic cover was treated as non-negotiable in our patient throughout the perioperative period.

Several practical principles emerge from this case. First, in an untreated high-grade AVM, the rate of sympathetic blockade matters as much as its depth; a graded epidural, titrated in small aliquots with haemodynamic assessment between doses, allows perfusion pressure to be held within a narrow range. Converting a percentage-based MAP target into an absolute trigger value in advance is a practical bedside strategy that speeds decision-making. Second, MAP, rather than systolic pressure, is the logical target because it determines cerebral perfusion pressure and aligns intraoperative and postoperative goals. Third, serial imaging is informative because these lesions are not always static; documented growth with new mass effect should raise the threshold for haemodynamic caution as pregnancy advances. Fourth, uninterrupted antiepileptic prophylaxis is essential because a perioperative seizure carries disproportionate risk in an unprotected nidus. Finally, thorough documentation of informed refusal provides the ethical and medicolegal basis for a conservative anaesthetic strategy when definitive treatment is declined. This case may serve as a basis for managing similar future presentations.

Limitations

First, the team did not obtain a full spinal MRI before neuraxial anaesthesia. Isikkent et al. recommend pre-procedural spinal imaging for any parturient with a known cerebral AVM to exclude spinal vascular malformations that could increase the risk of inadvertent puncture or haemorrhage during neuraxial access [11]. In this case, there were no clinical features of spinal vascular involvement (no radicular pain, bladder or bowel symptoms, or lower-limb neurological signs), and imaging resources were not available within the required time frame. We agree that the recommendation is reasonable where time and resources permit. A screening spinal MRI is planned at follow-up.

Second, this report describes a single-patient experience. Although the six-year imaging series and the favourable outcome under graded incremental epidural anaesthesia are informative, they do not establish that this technique is superior to alternative neuraxial approaches in this population. Comparative or registry-based data are needed before any technique can be considered the standard of care.

Third, the previous DSA and MRI image files could not be retrieved for figure reproduction. The findings from both studies are reported as documented in the original radiology reports, and only the latest MRI images were available for inclusion in the figure.

## Conclusions

In a parturient with an untreated S-M Grade V cerebral AVM, graded epidural anaesthesia achieved what mattered most: a controlled, predictable sympathectomy in a lesion that tolerates no sudden haemodynamic changes. The technique offers a neuraxial alternative where general anaesthesia has been the default. The accompanying six-year imaging series is a reminder that these lesions are not static and that, for women of reproductive age, the time to treat an AVM is before pregnancy, not during it.
